# Negative Regulation of Zap70 by Lck Forms the Mechanistic Basis of Differential Expression in CD4 and CD8 T Cells

**DOI:** 10.3389/fimmu.2022.935367

**Published:** 2022-07-04

**Authors:** Hassan Damen, Christian Tebid, Melissa Viens, Denis-Claude Roy, Vibhuti P. Dave

**Affiliations:** ^1^Institute for Hematology-Oncology, Cell and Gene Therapy, Hopital Maisonneuve-Rosemont Research Center, Montreal, QC, Canada; ^2^Department of Medicine, University of Montreal, Montreal, QC, Canada

**Keywords:** TCR signaling, Lck kinase, Zap70 kinase, negative regulation, CD4/CD8 T cells

## Abstract

Lck and Zap70, two non-receptor tyrosine kinases, play a crucial role in the regulation of membrane proximal TCR signaling critical for thymic selection, CD4/CD8 lineage choice and mature T cell function. Signal initiation upon TCR/CD3 and peptide/MHC interaction induces Lck-mediated phosphorylation of CD3 ITAMs. This is necessary for Zap70 recruitment and its phosphorylation by Lck leading to full Zap70 activation. In its native state Zap70 maintains a closed conformation creating an auto-inhibitory loop, which is relieved by Lck-mediated phosphorylation of Y315/Y319. Zap70 is differentially expressed in thymic subsets and mature T cells with CD8 T cells expressing the highest amount compared to CD4 T cells. However, the mechanistic basis of differential Zap70 expression in thymic subsets and mature T cells is not well understood. Here, we show that Zap70 is degraded relatively faster in DP and mature CD4 T cells compared to CD8 T cells, and inversely correlated with relative level of activated Zap70. Importantly, we found that Zap70 expression is negatively regulated by Lck activity: augmented Lck activity resulting in severe diminution in total Zap70. Moreover, Lck-mediated phosphorylation of Y315/Y319 was essential for Zap70 degradation. Together, these data shed light on the underlying mechanism of Lck-mediated differential modulation of Zap70 expression in thymic subsets and mature T cells.

## Introduction

T cells, an indispensable component of adaptive immunity, constantly patrol our body and eliminate pathogen infected or malignant cells *via* recognition by the T cell receptor (TCR) of antigenic peptide presented in association with the major histocompatibility complex (p/MHC) (1). TCR-induced signal transduction is essential for effective neutralization of such insults by mature T cells. TCR signaling is equally important for development of T cells in the thymus. It is implicated in maturation through the three distinct stages of thymocyte development: CD4^-^CD8^-^ double negative (DN), CD4^+^CD8^+^ double positive (DP) and CD4^+^ or CD8^+^ single positive (SP) thymocytes (2–4). Thymic selection of DP thymocytes ensures that thymocytes expressing high affinity TCRs are negatively selected while those with moderate/low affinity for self-p/MHC are positively selected and develop further into MHCII-specific CD4 helper and MHCI-specific CD8 cytotoxic T cells (5). In the periphery tonic TCR signaling induced by self-peptide/MHC play an important role in T cell homeostasis, while antigenic peptide/MHC complex induce differentiation into effector/memory T cells. Central to initiation and propagation of TCR signaling are two non-receptor tyrosine kinases, Lck, a Src family kinase, and Zap70, a Syk family kinase. The two kinases play an obligatory role in TCR signaling during thymic development and mature T cell function (6, 7). Lck is required for DN to DP transition as well as for thymic selection of DP thymocytes into SP thymocytes (2, 8–10). In contrast, Zap70 is critical for DP to SP transition although it is proposed to play some role in DN to DP transition and survival of DP thymocytes (11, 12).

Studies have shown that the cytoplasmic tails of CD4 and CD8 co-receptors serve as binding site for Lck (13, 14). Lck binds strongly to CD4 compared to CD8 cytoplasmic tail and engagement of CD4 mediates stronger Lck activation (15). The catalytic activity of Lck is tightly regulated by phosphorylation status of inhibitory Y505 at the C-terminus and activating Y394 located in the kinase domain. Phosphorylation by Csk kinase and dephosphorylation by CD45 phosphatase of the inhibitory Y505 in Lck promotes, respectively, closed inactive and open active conformation (16–23). A signal initiating step following TCR/pMHC engagement involves Lck-mediated phosphorylation of immunoreceptor tyrosine-based activation motifs (ITAM) of TCR-associated invariant CD3 chains (24, 25). Zap70 binds to phospho-CD3 *via* two tandem SH2 domains that permits Lck-mediated sequential phosphorylation of Y315/Y319 and Y493 leading to full Zap70 activation triggering downstream signaling cascade culminating in thymocyte or mature T cell activation (26, 27). Y315/Y319 play a critical role in maintaining the auto-inhibitory closed conformation of Zap70. Lck-mediated phosphorylation of Y315/Y319 relieves this auto-inhibition critical for full activation of Zap70 essential for TCR signaling. An essential role for Zap70 kinase is evident from impaired thymic selection in Zap70-deficient mice (28, 29).

While Lck is essential for DN to DP transition and thymic selection, it also modulates CD4 versus CD8 lineage choice of positively selected thymocytes. Augmented (inhibitory Y505F mutation) and diminished (kinase dead K273R mutation) Lck activity promotes CD4 and CD8 lineage choice, respectively, supporting the signal strength/duration model of CD4/CD8 lineage choice (30–32). Interestingly, studies using a mouse model of inducible Zap70 expression showed that sustained Zap70 expression is required for CD8 lineage choice although ablating Zap70 expression after signal transduction did not alter lineage choice (33–35). In WT mice Zap70 is expressed in very low amounts in DN thymocytes and its expression rises in DP and SP thymocytes, CD8 SP thymocytes expressing it particularly strongly (35). This pattern of Zap70 expression is opposite of Syk kinase expression, another member of the Syk kinase family, and the two kinases play unique roles in pre-TCR/TCR signaling during DN to DP transition (12, 36). Compared to mature T cells, DP thymocytes express substantially lower amount of TCR/CD3 complex, which is due to its rapid degradation upon induction of Lck activity following CD4 and MHCII interaction (15, 37, 38). Interestingly, in DP thymocytes Zap70 is associated with phosphorylated CD3ζ (39). Importantly, in DP thymocytes despite significant Lck activity induced by CD4/MHCII interaction TCR-associated Zap70 was shown to be inactive as judged by anti-phosphotyrosine Western blot (39). Zap70 expression is further elevated in SP thymocytes with CD8 T cells expressing highest amounts, yet this does not appear to be due to increased transcription suggesting post-translational regulation of Zap70 although the mechanism of this regulation is not understood.

In the present investigation, we show that under steady state relatively slower degradation explains the higher Zap70 expression in CD8 compared to CD4 SP and DP thymocytes. In addition, differential Zap70 expression in thymic subsets inversely correlated with relative amount of phospho-Zap70 levels in these cells. Augmenting Lck catalytic activity in a heterologous system and T cell line showed significant decrease in Zap70 expression. In transgenic mice expressing constitutively active Lck, we observed a severe diminution in Zap70 expression but a significant increase in relative amount of phosphorylated Zap70 in all thymic subsets and mature T cells. Further experiments showed that phosphorylation of Y315/Y319 of Zap70 by Lck played a critical role in Zap70 degradation. This study provides a strong evidence that Lck activity is a negative regulator of Zap70 expression, a finding with important implications for T cell development and function.

## Materials and Methods

### Mice

C57BL6 mice were bred in our animal facility. MHCI-restricted OTI^+^Rag^-/-^ (chicken ovalbumin 257-264 peptide specific) and P14^+^Rag^-/-^ (lymphocytic choriomeningitis virus (LCMV) GP_33-41_ peptide specific)) transgenic mice were obtained from Nathalie Labrecque and Heather Melichar (Centre de Recherche Hopital Maisonneuve-Rosemont (CRHMR)). MHCII^-/-^ mice were obtained from The Jackson Laboratory. All TCR transgenic mice were Rag-deficient unless mentioned otherwise. OTI mice expressing constitutively active Lck^Y505F^ or Thpok transgene were described previously (40). Mice were genotyped by peripheral blood analysis and/or PCR of genomic DNA isolated from an ear punch. Lymphoid organs from 5–8 week old mice were harvested for all analyses. All mice were housed under specific pathogen free conditions at the CRHMR. Animal experimentation protocols were approved by the CRHMR animal care committee and performed in accordance with the Canadian Committee for Animal Care.

### Transient Transfection

WT Lck and mutant expressing Y505F or K273R were cloned into pCCL lentiviral vector (kind gift of Jonathan Bramson, McMaster University). We also cloned Lck and mutants fused to mCherry into pCCL lentiviral vector (WT LCk-mCherry fusion kind gift of N. Gascoigne; National University of Singapore) (41). The Lck-mCherry fusion molecule expression is driven by an EF1α promoter. The plasmid also expresses truncated human NGFR under the control of CMV promoter. Lentivirus plasmid encoding full-length mouse Zap70 was obtained from Abm (pLenti-GIII-CMV-RFP-2A-Puro; cat # LV434826). In this plasmid Zap70 and RFP reporter expression were driven by CMV and SV40 promoter, respectively. RFP coding sequence was replaced by ZsGreen coding sequence (pLenti-Zap70-ZsGreen). Control lentiviral plasmid was generated by deleting Zap70 coding sequence (pLenti-ZsGreen). Y315F/Y319F (YFYFF) or Y319F mutated in pLenti-Zap70-ZsGreen were generated by overlap PCR. Mutations were confirmed by DNA sequencing. One million 293T cells were co-transfected with plasmids encoding mouse Zap70 and pCCL plasmids expressing control or Lck variants in the presence of polyethylenimine (PEI). Ninety-six hours post transfection cells were fixed, permeabilized and stained for Zap70 followed by flow cytometry analysis. Mouse Zap70 expression was determined in ZsGreen+NGFR+ cells and normalized to Zap70 expression ZsGreen+ cells transfected with control plasmid for Lck expression. Plasmid encoding human Zap70-GFP fusion molecule linked to EGFR by self-cleaving T2A peptide sequence was obtained from Johannes Huppa (Medical University of Vienna) (42) and a hZap70-GFP expressing 293T stable cell line was generated. This stable cell line was transfected with various Lck-mCherry fusion lentivirus constructs. For human Zap70-GFP fusion protein expression analysis, GFP expression in mCherry expressing or not cells was analyzed by flow cytometry and expressed as ratio of mCherry-/mCherry+ cells. In some experiments Zap70-GFP expressing 293T cells were transfected in triplicate with NGFR reporter expressing control, Lck^WT^ or Lck^Y505F^ plasmids and stained intracellularly with PE-conjugated anti-pY319 antibody. Ratio of MFI for pY319 over GFP in NGFR+ve and NGFR-ve cells was determined and normalized to control transfectants.

### Lentivirus Production and Transduction

For lentivirus production 293T cells were plated in 10cm tissue culture dish and transfected with Lck^WT^-mCherry, Lck^Y505F^-mCherry or Lck^K273R^-mCherry lentiviral plasmid and pMD2.G and psPAX2 packaging plasmids (Addgene) in the presence of polyethyleneimine. Culture supernatant containing lentiviral particles was harvested at 48 and 72 hours post-transfection and used to transduce the RLM mouse CD4 SP thymocyte cell line. Zap70 expression in transduced cells (mCherry+) was analysed using flow cytometry.

### Flow Cytometry

A total of 1 x 10^6^ thymocytes or RBC-depleted spleen cells were incubated with a combination of fluorescently labeled Abs to CD4 (GK1.5), CD8 (53-6.7), TCRβ (H57-957), CD5 (53-7.3), CD69 (H1.2F3), CD24 (M1/69), CD44 (IM7), CD62L (MEL-14), Vα2 (B20.1), Vβ5 (MR9-4), and analyzed using flow cytometry (LSRFortessa X-20 or LSR II, BD Biosciences). For intracellular labeling thymocytes or spleen cells were surface stained, fixed and permeabilized using commercial kit (Ebioscience, FoxP3 fixation/permeabilization buffer) and stained for Lck, Zap70, pY319-Zap70, and pY493-Zap70. Appropriate isotype control antibody staining was included in all experiments involving intracellular staining. Antibodies were obtained from eBioscience, BioLegend, or Cell Signaling Technology. Data were analyzed using FlowJo software (Tree Star).

### Quantitative RT-PCR

Various thymic or splenic T cell subsets were FACS purified and total RNA isolated using TRIzol (Invitrogen). Complementary DNA was synthesized using a commercial kit as per manufacturer’s protocol (Bio-Rad Laboratories). Quantitative PCR (QPCR) for target gene was performed in triplicate using SYBER green dye (Bio- Rad Laboratories) or EvaGreen dye (Abcam). Amplification of housekeeping gene Hprt served as an internal control. QPCR data were analyzed by Applied Biosystems software ABI 7500 v2.0.5. Data were normalized to Hprt expression in each population. Relative expression values were calculated using ΔΔ cycle threshold method. Ratio of gene-specific values to housekeeping gene for each subset was determined and normalized to CD4 T cells for splenic T cell analysis and to DN thymocytes for thymic subset analysis. Data are presented as an average of triplicate values with standard deviation (SD). QPCR primers for Hprt, Lck and Zap70 were obtained from Integrated DNA Technologies.

### Kinetics of Zap70 Expression

To determine the kinetics of Zap70 degradation splenic T cells were isolated using negative selection beads (Stem Cell Technology) followed by FACS sorting to obtain purified CD4 and CD8 T cells. One million CD4 and CD8 T cells were cultured in the presence of cyclohexamide, an inhibitor of protein translation, for different time points followed by lysis in Laemmeli buffer. Lysates were resolved on polyacrylamide gel under reducing condition, transferred onto PVDF membrane, blocked in 5% milk in TBST buffer (10mM Tris pH7.6, 150mM NaCl and 0.1% Tween20) for 1 hour at room temperature followed by anti-Zap70 or anti-actin antibody overnight (O/N) at 4°C. Membranes were washed four times with 0.1%TBST and further incubated with HRP coupled secondary antibody, washed four times with 0.1%TBST, and developed using chemiluminescent reagent. Bands were visualized using Azure c600 imaging system and their intensity determined using ImageJ software. Ratio of band intensity of Zap70 over actin was determined and normalized to that at 0 minute as 100%. To study the effect of activation on Zap70 degradation one million purified CD4 and CD8 T cells were incubated with biotinylated anti-CD3 antibody (10μg/ml) for 30 minutes on ice, washed with ice-cold media and stimulated by adding pre-warmed media containing streptavidin (20μg/ml) for 5, 10, 60 and 120 minutes. Cell were processed for Western blot as described above. Incubation, wash and stimulation media contained cyclohexamide. For the analysis of Zap70 degradation in thymocytes, cells were cultured in the presence of cyclohexamide, surface stained for CD4/CD8/TCR followed by Zap70 intracellular staining at 30 min, 1, 2, and 4 hr post-incubation. To account for variability in staining we determined and compared the ratio of Zap70 expression in target subset over DN thymocytes (which express low amount of Zap70); the ratio for DN thymocytes was treated as one. To determine Zap70 ubiquitination purified T cells from OTI mice expressing or not Lck^Y505F^ transgene were lysed and anti-Zap70 immunoprecipitate was Western blotted with anti-Ubiquitin or anti-Zap70 antibody, and the ratio of anti-Ub to anti-Zap70 band intensity was compared.

### Statistical Analyses

Statistical analyses were performed using GraphPad or Microsoft Office. Excel software. Data are displayed as a mean with SD error bar. Unpaired two-tailed Student t test was used to determine statistical significance when thymic and splenic T cell subsets from different mice. For experiments involving a comparison of T cell subsets isolated from the same mouse, a paired Student t test was used. A *p* value < 0.05 was considered statistically significant (*p < 0.05, **p < 0.005, *p* ***< 0.0005 and ****p < 0.00005).

## Results

### Zap70 Expression Is Inversely Correlated With Its Activation Status

**Figure 1:**
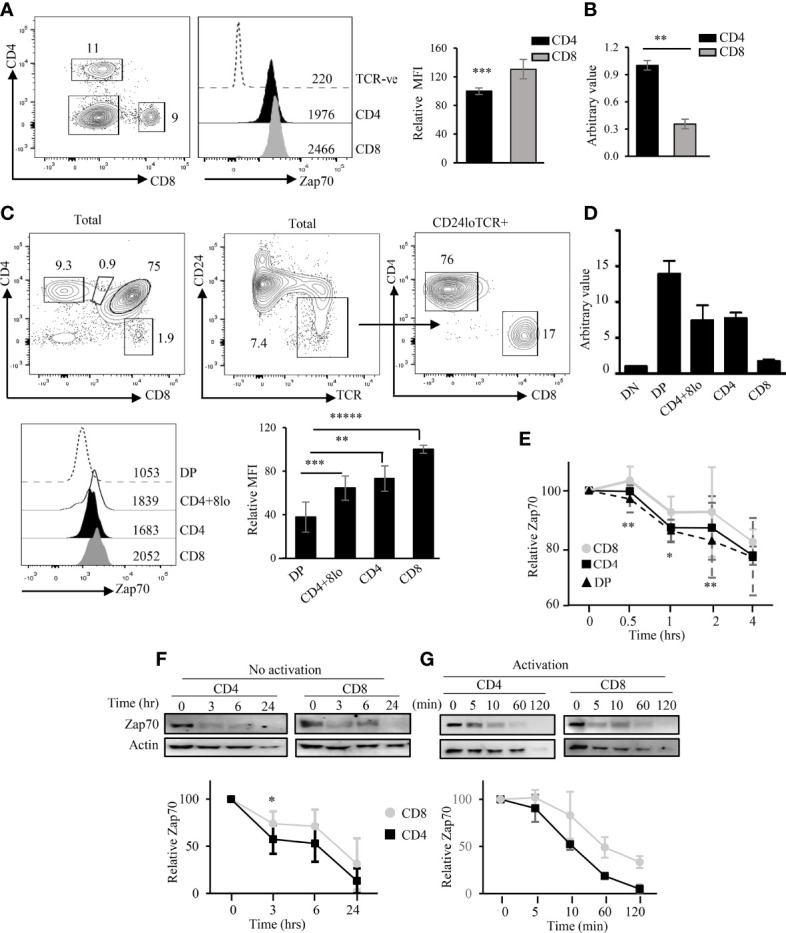
Differential Zap70 stability in thymic and splenic T cells. **(A)** shows a representative example of CD4/CD8 staining and Zap70 histogram of splenic CD4 and CD8 T cells from WT mice and bar graph comparing Zap70 mean fluorescent intensity (MFI) normalized to Zap70 expression in CD8 T cells (n=16 mice in 6 or more independent experiments). Staining in TCR^-ve^ cells serves as control for Zap70 specific staining. **(B)** QPCR analysis of Zap70 transcript in purified WT splenic CD4 and CD8 T cells. Average of triplicate for two biological replicate and SD is shown. Data normalized to Zap70 value in CD4. **(C)** Zap70 staining for DP and CD4^+^CD8^lo^ (gated for total thymocytes) and CD4^+^ and CD8^+^ SP thymocytes (gated for CD24^lo^TCRβ^hi^) thymocytes is shown. Bar graph shows compilation of Zap70 MFI for various thymic subsets normalized to Zap70 MFI in CD8 SP thymocytes (n ≥ 10 in 6 or more independent experiments). **(D)** Zap70 transcription in purified thymic subsets was determined by QPCR and normalized to that in DN thymocytes. An average of triplicate for two biological replicate and SD is shown. **(E)** Kinetics of Zap70 expression in thymocytes was determined by flow cytometry. Thymocytes were cultured in the presence of cyclohexamide for the indicated time point, surface stained followed Zap70 intracellular stain. MFI for each time point was normalized to 0 hr time point. Kinetics of Zap70 protein degradation in unactivated **(F)** and activated **(G)** purified CD4 and CD8 T cells in the presence of cyclohexamide was determined by Western blot. Graph shows ratio of Zap70/actin band intensity for each time point normalized to 0 hr time point. Data representative of two independent experiments **(B, D, E, G)** or three experiments **(F)**. Frequency of cells and MFI values are shown in FACS plots and histograms **(A, C)** *p < 0.05, **p < 0.005, ***p < 0.0005, ****p < 0.00005, *****p < 3.9 × 10-6 (10 to power -6).

To dissect the mechanism of differential Zap70 expression in CD4 and CD8 T cells or thymic subsets, we first evaluated Zap70 expression in cells from wild type (WT) mice. To this end, we first surface stained splenocytes for CD4, CD8 and TCR followed by fixation, permeabilization and intracellular staining for Zap70. This highly sensitive approach allowed us to accuarately evaluate Zap70 expression at the single cell level. Here, we observed a significantly higher level of Zap70 protein in mature CD8 than CD4 splenic T cells despite higher Zap70 mRNA expression in the latter subset (**Figures 1A,B**; **Figure S1**), which is in agreement with published report (35). Similar analysis of WT thymocytes showed a direct correlation between thymocyte maturation and Zap70 expression with CD8 SP and DP thymocytes expressing the highest and lowest Zap70 amount, respectively (**Figure 1C**) (12, 35). Further, CD4^+^CD8^lo^ and CD4^+^ SP thymocytes expressed significantly more Zap70 than DP thymocytes but less than CD8 SP thymocytes. Interestingly, at the transcriptional level, higher Zap70 mRNA levels were observed in DP compared to more mature CD4^+^CD8^lo^ or CD4^+^ and CD8^+^ SP thymocytes (**Figure 1D**). These data suggest differential Zap70 stability in DP thymocytes and in CD4 mature thymocytes compared to CD8 thymocytes or mature T cells. To test this notion, we evaluated the kinetics of Zap70 degradation in thymocytes and mature T cells in the presence of cyclohexamide, a protein translation inhibitor. Indeed, flow cytometric analysis of Zap70 expression kinetics of WT thymocytes showed faster degradation in DP and CD4 SP thymocytes compared to CD8 SP thymocytes (**Figure 1E**). Similarly, anti-Zap70 Western blot analysis of mature CD4 and CD8 T cells showed faster Zap70 degradation in CD4 T cells compared to CD8 T cells; at 180 minutes Zap70 expression was reduced by 60% in CD4 T cells compared to about 35% in CD8 T cells (**Figure 1F**). We then determined that T cell activation resulted in accelerated Zap70 degradation with CD4 T cells showing higher degradation compared with CD8 T cells (**Figure 1G**) and is in agreement with a previous report (43). These data support the notion that Zap70 is relatively less stable in DP thymocytes and CD4 SP thymocytes compared to CD8 SP thymocytes or T cells.

**Figure 2:**
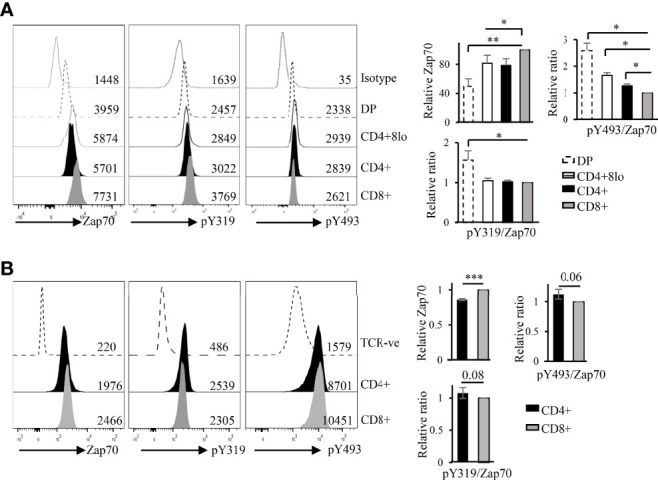
Zap70 stability inversely correlates with relative phosphorylation of the protein in thymocytes and mature T cell subsets. Histogram shows a representative example of phospho staining of thymic **(A)** and splenic **(B)** T cell subsets from WT mice. Bar graph shows compilation of relative amount of total Zap70 and pY493 and pY319 Zap70 (expressed as ratio of phospho MFI over total Zap70 MFI) normalized to that in CD8 SP thymocytes or CD8 splenic T cells. MFI values are shown in the histograms (6 mice from 3 independent experiments) *p < 0.05, **p < 0.005, and ***p < 0.0005.

A previous report showed, by Western blot, that non-phosphorylated Zap70 is associated with a substantial fraction of surface TCR in the preselection DP thymocytes *via* phospho-CD3ζ (39). As our data and published report show that DP thymocytes express the lowest amount of Zap70, and since recruitment of Zap70 to phospho-CD3ζ should promote its phosphorylation we asked whether Zap70 levels correlated with its phospho status. To this end, we used flow cytometry to determine relative amounts of phospho Y319 (pY319) and pY493 forms of Zap70, which represent partially and fully active kinase, respectively. As Zap70 is differentially expressed in the thymic and splenic subsets, we determined relative amounts of phophos-Zap70 by calculating the ratios of the two phospho forms of Zap70 to the total amount of Zap70 in each thymic or splenic T cell subset and normalized it to the average of ratio in CD8 SP thymocytes or mature T cells. Surprisingly, we observed that the relative amount of pY319 and pY493 Zap70 expression correlated inversely with the total Zap70 expression. Thus, DP thymocytes expressed significantly higher relative amount of pY319 and pY493 Zap70 than post-selection mature thymocyte subsets (**Figure 2A**). Further, CD4 SP thymocytes showed higher relative pY493 levels than CD8 SP thymocytes. Similar analysis of splenocytes showed higher relative amount of pY319 and pY493 Zap70 in CD4 compared to CD8 T cells although the difference was not significant (**Figure 2B**). Together, the inverse correlation between total Zap70 level and its phosphorylation suggest that partial and/or full Zap70 activation may negatively affect its stability.

### Zap70 Expression Is Negatively Regulated by Lck Activity

**Figure 3:**
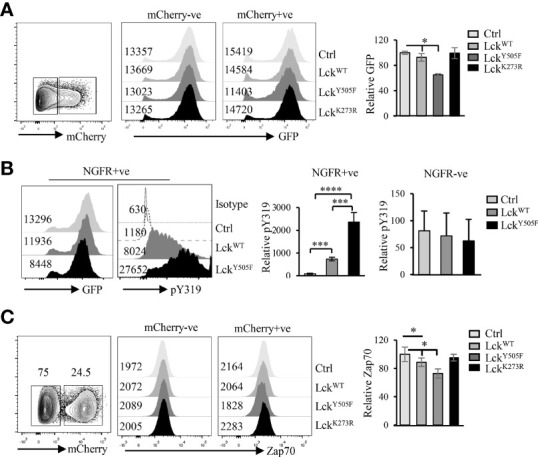
Elevated Lck activity negatively affects Zap70 expression. **(A)** 293T cells stably expressing hZap70-GFP fusion were transfected with plasmid expressing control, WT-Lck, constitutively active Lck^Y505F^ or kinase-dead Lck^K273R^ fused to mCherry. Histograms show GFP expression in mCherry-ve and mCherry+ve cells. Bar graph shows average of ratio of GFP expression in mCherry+/mCherry- cells normalized to that in control transfectants for three independent experiments. **(B)** Histogram shows a representative GFP expression and pY319 Zap70 staining in NGFR+ve 293T cells transfected with control, Lck^WT^ or Lck^Y505F^ plasmids. Bar graph shows an average of ratio of MFI for pY319 to GFP normalized to that for control transfectants in NGFR+ve and NGFR-ve cells from two independent experiments. **(C)** CD4 SP thymocyte cell line RLM was transduced with lentivirus expressing control, WT-Lck, constitutively active Lck^Y505F^ or kinase-dead Lck^K273R^ fused to mCherry. Zap70 expression in RLM cells expressing or not mCherry was determined. A representative histogram for Zap70 staining in mCherry- and mCherry+ cells is shown. Bar graph shows average of ratio of mCherry+/mCherry- cells normalized to control for three independent experiments. Error bar represents S.D *p < 0.05, ***p < 0.0005, ****p < 0.00005.

It is well established that Lck is more active in CD4 than CD8 T cells (15, 44). As Lck phosphorylates Y319 and Y493 in Zap70 (45, 46) and data described above suggest an inverse correlation between phospho status of Zap70 and its total amount, we asked if differential Lck activity was the cause of differential Zap70 expression in CD4 and CD8 T cells. To test this prediction, we generated constitutively active Lck^Y505F^ and kinase inactive Lck^K273R^ mutants. In these constructs Lck was fused to mCherry allowing us to monitor Lck expression by analyzing mCherry expression (41). We also generated 293T cells stably expressing human Zap70-fused to GFP (hZap70-GFP). This system allowed us to determine Zap70 expression by monitoring GFP levels (42). We transfected hZap70-GFP expressing 293T cells with Lck-mCherry fusion constructs and monitored GFP expression in mCherry expressing cells to determine the impact of Lck activity on Zap70 expression. Indeed, we observed significant decrease in Zap70 expression in the presence of Lck^Y505F^ (~30% decrease in GFP) compared to control or WT Lck, while it was unaltered in the presence of kinase inactive Lck^K273R^ mutant (**Figure 3A**). A similar decrease in mouse Zap70 expression was observed in the presence of Lck^Y505F^ compared to WT Lck in 293T cells (data not shown). Importantly, we observed an opposite patterns for pY319 Zap70 staining; WT Lck expression resulted in significant increase in pY319 Zap70 level compared with basal level in control transfectants, and this amount increased by 3-fold in the presence of Lck^Y505F^ (**Figure 3B**). To extend this observation to T cells we we transduced RLM, a CD4 SP thymocyte cell line, with lentivirus expressing WT or Y505F or K273R mutated Lck fused to mCherry and analyzed endogenous Zap70 expression by flow cytometry in mCherry+ve and mCherry-ve RLM cells. As expected, CD4 cell line expressing constitutively active Lck^Y505F^ showed significant decrease in Zap70 levels compared to those expressing WT Lck or kinase inactive Lck^K273R^ (**Figure 3C**). Zap70 expression was comparable in untransduced mCherry-ve RLM cells in all conditions. These data strongly suggest an inverse correlation between Zap70 amount and its activation state

**Figure 4:**
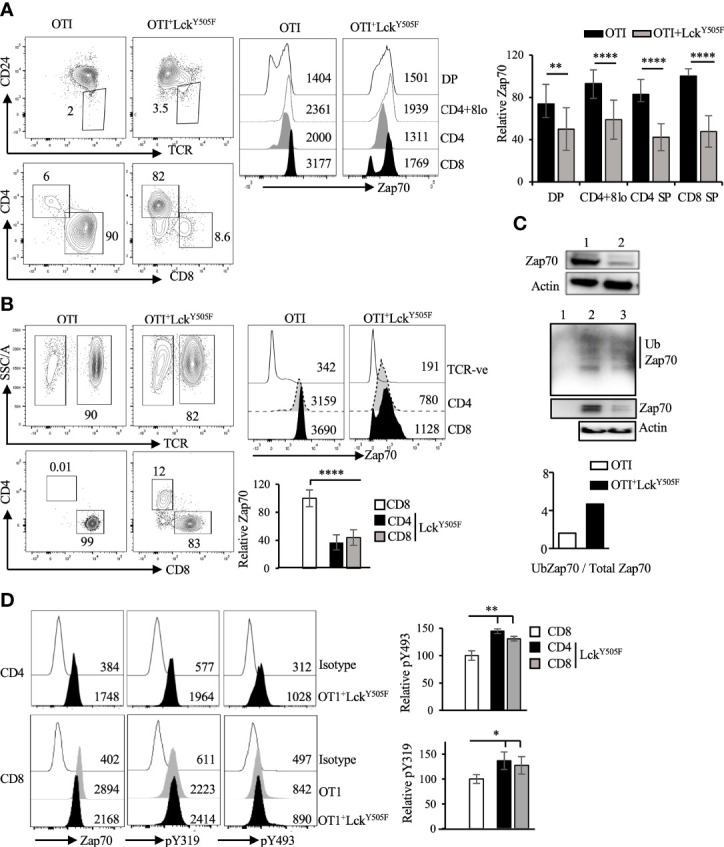
Constitutively active Lck results in Zap70 diminution *in vivo*. **(A)** shows CD24/TCR staining of total thymocytes and CD4/CD8 profile of mature thymocytes (CD24^lo^TCR^+^) from the indicated mice. Histograms show Zap70 staining and bar graph shows comparison of relative Zap70 expression in DP, CD4^+^CD8^lo^, CD4^+^ and CD8^+^ thymocytes from OTI mice expressing or not Lck^Y505F^ transgene. Zap70 expression was normalized to that in CD8^+^ thymocytes from OTI control mice in each experiment. **(B)** CD4/CD8 profile and Zap70 staining of CD4 and CD8 T cells from OTI mice expressing or not Lck^Y505F^ is shown. Bar graph shows compilation of Zap70 MFI in the indicated T cell subsets from OTI and OTI^+^Lck^Y505F^ mice normalized to that in CD8 T cells from OTI mice in each experiment. **(C)** Western blot shows a representative example of Zap70 and actin expression in total lysates of purified T cells from control (lane 1) and Lck^Y505F^ (lane 2) expressing OTI mice. Middle panel shows anti-Ubiquitin Western blot of Zap70 immunoprecipiates of purifies splenic T cells from OTI (lane 2) and OTI^+^Lck^Y505F^ (lane 3) mice. Lane 1 is anti-Zap70 antibody control. Total lysates was probed for actin and serves as control for cell numbers. (n=2 independent experiments). **(D)** Histograms for total, pY319 and pY493 Zap70 staining in CD4 and CD8 T cells from the indicated mice is shown. Bar graph shows relative pY319 and pY493 value expressed as ratio of MFI for phospho staining to total Zap70 staining for CD4 and CD8 T cells from OTI^+^Lck^Y505F^ mice (filled bars) and normalized to that in CD8 T cells from OTI mice (open bar). Data are representative example of 6 **(A, B)** or 3 **(D)** independent experiments. Numbers in FACS plots and histograms represent frequency of cells and MFI values *p < 0.05, **p < 0.005, and ****p < 0.00005.

**Figure 5:**
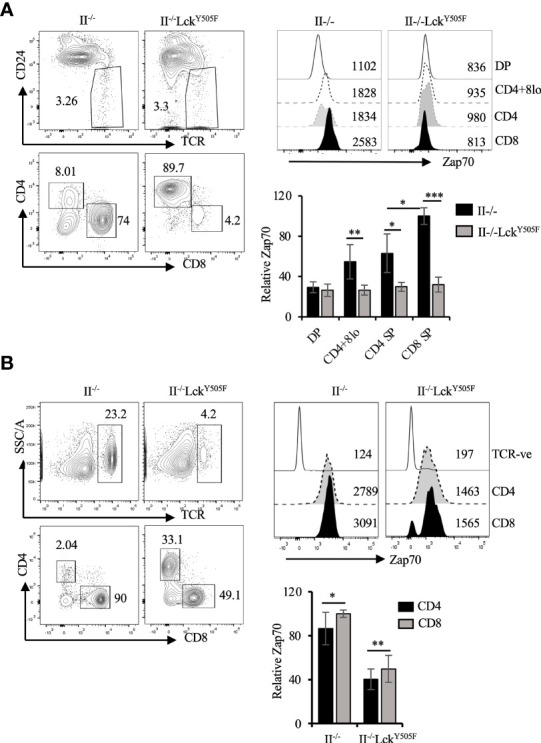
Diminution of Zap70 expression in thymocytes and mature T cells in MHCII^-/-^ mice expressing Lck^Y505F^ transgene. **(A)** CD24/TCR and CD4/CD8 staining of, respectively, total and mature (CD24^lo^TCR^hi^) thymocytes is shown. Histograms show Zap70 staining and bar graph compares relative Zap70 expression in the indicated thymic subsets from MHCII^-/-^ mice expressing or not Lck^Y505F^. **(B)** shows TCR and CD4/CD8 staining of splenic T cells, histograms for Zap70 staining and data compilation for relative Zap70 expression in CD4 and CD8 T cells from MHCII^-/-^ mice expressing or not Lck^Y505F^ transgene. Data are representative example of 4 or more independent experiments (n > 6 mice). *p < 0.05, **p < 0.005, ***p < 0.0005.

To provide *in vivo* evidence for this inverse correlation between Lck activity and Zap70 expression, we took advantage of mice expressing constitutively active Lck transgene (Lck^Y505F^), which promotes redirection of MHCI-signaled thymocytes into CD4 lineage (10, 31). In addition, we recently showed that expression of Lck^Y505F^ transgene augments Thpok-induced CD4 lineage choice of MHCI-signaled thymocytes (40). We therefore asked if Zap70 expression was altered in T cells from MHCI-specific OTI-TCR transgenic mice expressing Lck^Y505F^. In agreement with our ex vivo data, we observed a dramatic reduction in Zap70 expression, as much as 70%, in the thymic subsets (**Figure 4A**) as well as mature CD4 and CD8 splenic T cells from OTI^+^Lck^Y505F^ mice compared to OTI control mice (**Figure 4B**). This was confirmed by anti-Zap70 Western blot of splenic T cells from OTI^+^Lck^Y505F^ and OTI control mice (**Figure 4C**). Anti-ubiquitin Western blot of anti-Zap70 immunoprecipitates of purified splenic T cells showed higher relative amounts of ubiquitinated Zap70 in purified splenic T cells from OTI^+^Lck^Y505F^ compared to OTI control (**Figure 4C**). Interestingly, CD4 T cells consistently showed lower Zap70 expression compared to CD8 T cells from OTI^+^Lck^Y505F^ mice although it was not significant (**Figures 4A,B**). Despite the decrease in total Zap70 the relative amounts of pY493 and pY319 were significantly increased in mature T cells from OTI^+^Lck^Y505F^ mice compared to those in CD8 T cells from OTI mice (**Figure 4D**) supporting the idea that Zap70 expression inversely correlates with its activation status. Decrease in Zap70 expression thymocytes and mature T cells from MHCII^-/-^ (**Figure 5**) and P14 (**Figure S2**) mice expressing Lck^Y505F^ compared to control mice confirmed these data. Interestingly, we found that a small number of CD4 T cells that develop in MHCII^-/-^ mice also showed significantly lower Zap70 expression compared to CD8 T cells (**Figure 5**) (both restricted to MHCI) providing further evidence for a role of the co-receptor associated Lck in regulating Zap70 expression.

**Figure 6:**
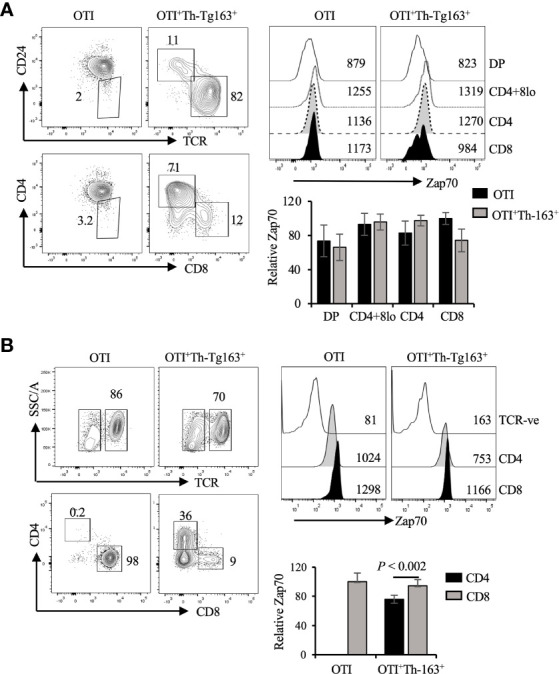
Constitutive Thpok expression does not alter Zap70 expression. **(A)** CD24/TCR and CD4/CD8 staining of, respectively, total and mature thymocytes and Zap70 histograms for the indicated thymic subsets from OTI and OTI^+^Th-163^+^ mice is shown. Bar graph shows comparison of relative Zap70 MFI in the indicated thymic subsets from OTI mice expressing or not Th-163 transgene. **(B)** shows TCR and CD4/CD8 staining of splenic T cells and Zap70 expression in CD4 and CD8 T cells from OTI and OTI^+^Th-163^+^ mice. Bar graph shows relative Zap70 expression in CD4 and CD8 T cells from the indicated mice (n>4 mice in three independent experiments). Frequency of cells and MFI values are shown in FACS plots and histograms.

To ensure that the decrease in Zap70 expression was due to Lck^Y505F^ expression rather than lineage redirection we performed Zap70 staining of thymocytes and mature T cells from OTI mice expressing the Thpok transgene. We previously showed that enforced Thpok expression promotes CD8 to CD4 lineage redirection in dose dependent manner and is augmented by Lck^Y505F^ expression (40). Thus, we analyzed Zap70 expression in mice expressing two different Thpok transgenes, Thpok-163 and Thpok-H. We detected a small decrease in Zap70 levels in the redirected CD4 T cells while it remained unchanged in CD8 T cells from OTI mice expressing Thpok-163 (**Figure 6**) and Thpok-H transgene (**Figure S3**). Together, these data strongly suggest that Lck activity, while it augments Zap70 phosphorylation, negatively impacts the stability of Zap70 in SP thymocytes and mature T cells, and explain the lower Zap70 expression in CD4 compared to CD8 T cells in wild type mice.

### Phosphorylation of Zap70 Y315/Y319 Contribute to Lck-Induced Zap70 Degradation

**Figure 7:**
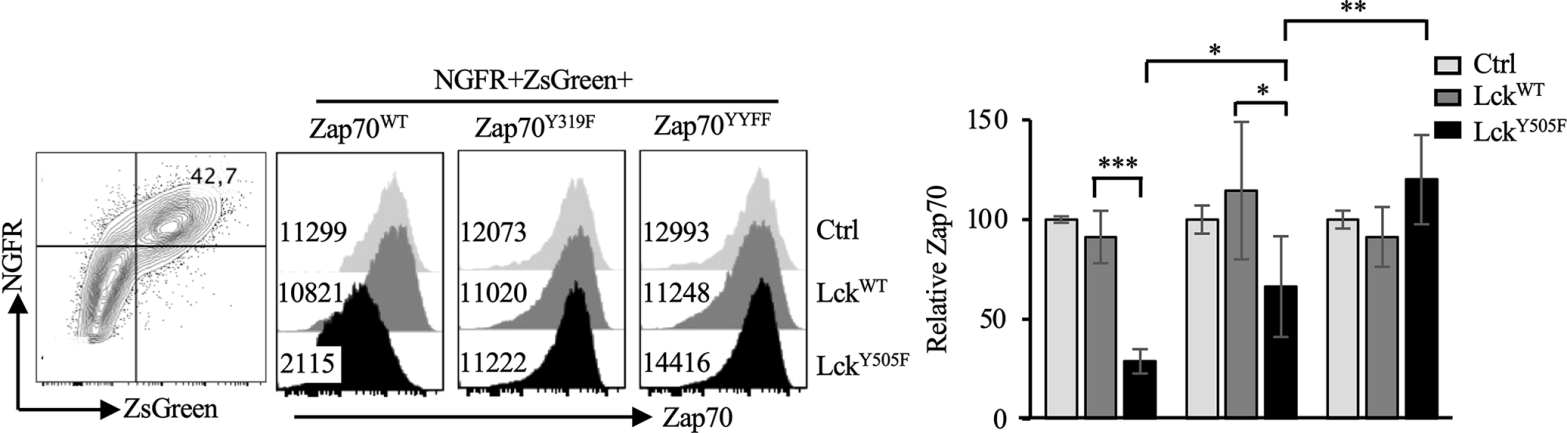
Phosphorylation of Y315/Y319 in Zap70 is required for Lck-induced Zap70 degradation. 293T cells were co-transfected with control, Lck^WT^ or Lck^Y505F^ (expressing NGFR reporter) and Zap70^WT^, Zap70^Y319F^ or Zap70^YYFF^ (expressing ZsGreen reporter) plasmids. Histograms show Zap70 expression in NGFR+ZsGreen+ 293T cells. Bar graph shows compilation of relative Zap70 expression in the indicated 293T transfectants from three independent experiments. *p < 0.05, **p < 0.005, ***p < 0.0005.

In its inactive form Zap70 is auto-inhibited by intra-molecular interactions between interdomain A (linking the two SH2 domains) and kinase domain mediated by Y315 and Y319 located in the interdomain B (linking C-SH2 to kinase domain). Lck phosphorylates Y315/Y319 in the interdomain B relieving auto-inhibition and leading to an open conformation of Zap70 that is partially active. Doubly phosphorylated pY315/pY319 Zap70 serves as a docking site for Lck *via* its SH2 domain causing phosphorylation of Y493 in the kinase domain and full activation of Zap70. Data described above strongly suggest an inverse correlation between Lck activity and Zap70 expression. Interestingly, Y315A/Y319A (YYAA) knockin mutations, which result in an open conformation, generate a hypomorphic Zap70 allele and impairs thymic selection and mature T cell function in mice (47). Lck cannot bind to YYAA Zap70 mutant although the mutations promote auto-phosphorylation and LAT phosphorylation (48). This suggests that relieving auto-inhibitory constrain by phosphorylation of Y315/Y319 in Zap70 may induce its degradation. To test this notion we generated and co-transfected WT, Y319F or YYFF mutated Zap70 (also expressing ZsGreen reporter) with WT or constitutively active Lck^Y505F^ (expressing NGFR reporter). The YYFF mutated Zap70 cannot be phosphorylated by Lck, thus resulting in a constitutively closed auto-inhibitory structure that impairs TCR signaling and affects T cell development and function with Y319F, compared to Y315F, mutated Zap70 having more profound impact on T cell function (46, 48–50). Taking advantage of YYFF or Y319F mutated Zap70, we analyzed and compared Zap70 expression in NGFR+ZsGreen+ cells. As predicted, constitutively active Lck^Y505F^ had no effect on Zap70^YYFF^ expression but resulted in severe reduction in Zap70^WT^ expression. Interestingly, Lck^Y505F^ induced significantly less degradation of Zap70^Y319F^ compared to Zap70^WT^ but more compared to Zap70^YYFF^ mutant (**Figure 7**). These data provide substantial evidence that relieving auto-inhibition *via* Lck-mediated phosphorylation of Y315/Y319 renders Zap70 considerably unstable.

## Discussion

**Figure 8:**
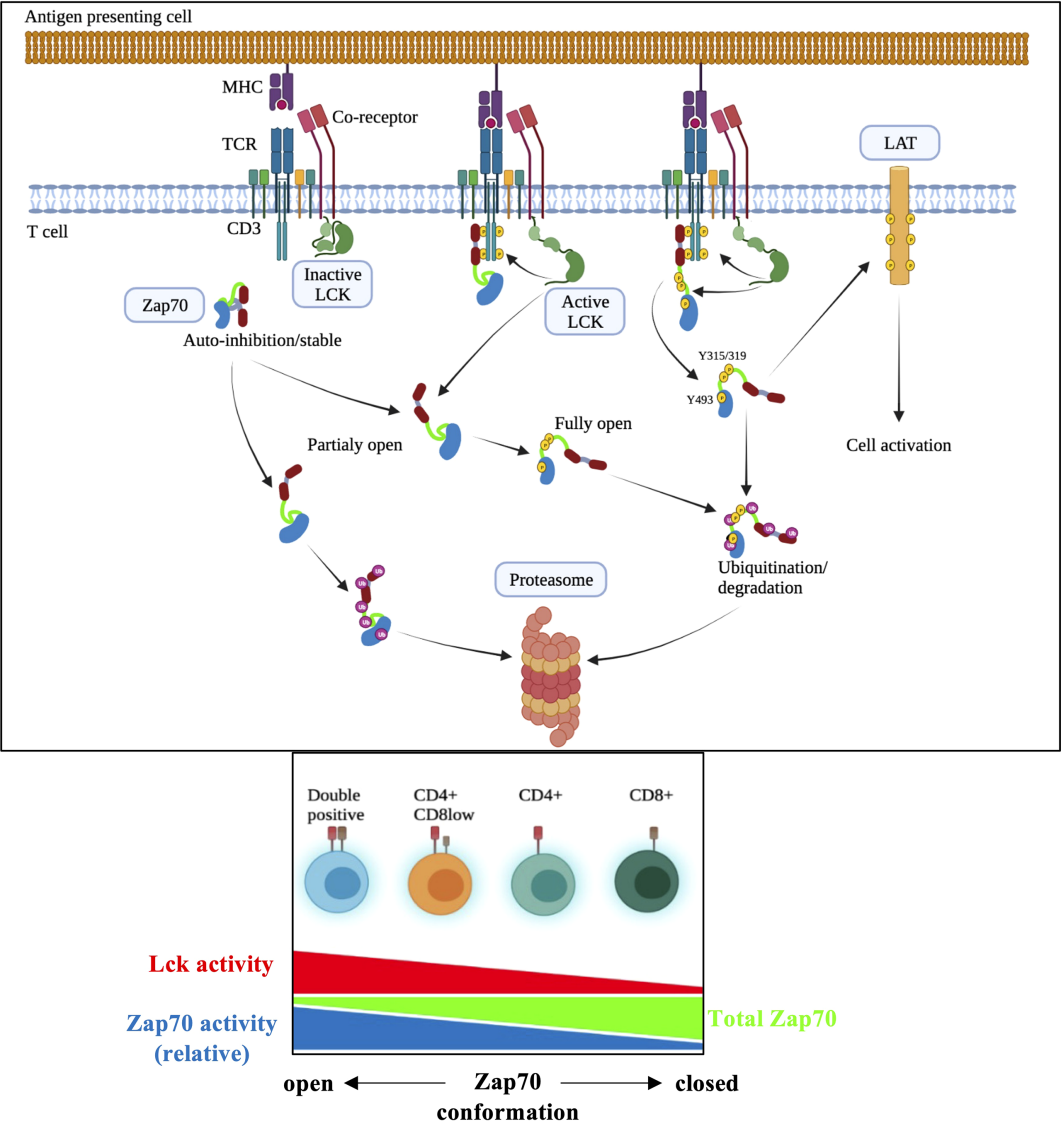
Model of Lck-mediated regulation of Zap70 expression/activation in T cell activation. In DP thymocytes higher amount of Zap70 is associated with surface TCR complex compared with mature T cells (ref 39) likely due to higher Lck activity resulting from CD4 and MHCII interaction (ref 15). We propose that the closed auto-inhibited conformation of Zap70 is stable and upon its recruitment to phosphor-CD3 chains Lck-mediated phosphorylation of Y315/Y319 and Y493 results in partially or fully active Zap70 essential for signal transduction during thymic maturation and mature T cell activation. However, partially or fully active Zap70 must be rapidly degraded to prevent potentially dominant negative effect of partially active Zap70 or over-activation during thymic development or mature T cell response to ligand. Partially or fully open but free (not associated with phospho-CD3) Zap70 may be rapidly degraded as well thereby preventing undesired antigen independent signaling during thymic development. It is quite possible that association of partially open Zap70 with phospho-CD3 may protect it from degradation.

Lck and Zap70 kinases are crucial for proximal TCR signaling that forms the basis of thymic selection and CD4/CD8 lineage choice in the thymus and mature T cell homeostasis and response to antigenic challenge. While Lck is more active in CD4 than CD8 T cells (15, 44, 51), higher Zap70 levels are found in CD8 than CD4 T cells (35). Here, we have studied the mechanistic basis of differential Zap70 expression in correlation to Lck activity in thymic subsets and mature T cells employing flow cytometry staining that allows analysis at single cell level. Our data show that Lck-mediated phosphorylation of Y315 and Y319 in the interdomain B, which results in an open active conformation, is sufficient to induce rapid Zap70 degradation. Apart from its role in regulating TCR expression *via* CD3 ITAM phosphorylation and serving as a bridge between Zap70 and its substrate LAT (15, 52, 53), our finding that Lck kinase function negatively regulates Zap70 expression identifies a novel function for Lck and may explain several aspects of T cell biology (**Figure 8**). Given that the two kinases are essential for proximal events in signal transduction in all T cells (αβ, γδ, NKT cells) this simple mechanism of regulating Zap70 expression by Lck may regulate TCR signaling in developing thymocytes in response to selecting ligands critical for production of mature T cells devoid of autoimmunity and in mature T cells in response to self or foreign peptide.

In the thymus Zap70 expression inversely correlates with thymocyte maturation with expression in DP < CD4 < CD8 thymocytes with DP thymocytes expressing about 2.5 to 3-fold less Zap70 than CD8 SP thymocytes, and lower Zap70 expression in CD4 than CD8 SP thymocytes was retained in mature T cells with CD4 expressing lower Zap70 than CD8 T cells. Analysis of relative pY493 and pY319 specific staining showed exactly opposite pattern with higher relative expression in DP thymocytes compared to SP thymocytes. Previous study showed that non-phosphorylated Zap70 is associated with phopho-CD3 chain in the preselection DP thymocytes (39, 54). It is possible that lower amount of Zap70 in DP thymocytes combined to anti-pY Western blot technique likely resulted in lack of detection of pY-Zap70 in DP thymocytes in these studies. This opposite pattern of total and relative phospho-Zap70 levels prompted us to evaluate role of Lck-mediated phosphorylation of Zap70 in regulating Zap70 expression. If non-phosphorylated Zap70 remains associated with phosho-CD3 chains it may act as a dominant negative (55) leading to impaired signaling in the preselection DP thymocytes. Lck-mediated phosphorylation of Y315/Y319 in Zap70 in DP thymocytes may serve two functions; in the absence of optimal receptor/ligand interaction it may lead to dissociation of Zap70 from TCR complex (56) and its subsequent degradation thereby preventing its potential dominant negative effect on TCR signaling. Such a mechanism may help explain sensitive nature of DP thymocytes to selecting self-peptide ligands (57); the “primed” Zap70 readily available for full activation upon engagement of DP thymocytes with a selecting ligand. However, we do not rule out the possibility that association of non-phosphorylated Zap70 to phospho-CD3 may protect it from degradation and that partially or fully open but free Zap70 is targeted for degradation in DP thymocytes thereby preventing undesired signal transduction in unsignaled DP thymocytes. Equally, fully Zap70 activation upon signal transduction in DP thymocytes, while necessary for efficient signaling, may cause over activation if its levels are not controlled; degradation would then regulate the amount of fully active Zap70 available for signaling (**Figure 8**).

Our data also explains paradox that sustained Zap70 expression is required for CD8 lineage choice, while augmented Lck activity promotes CD4 lineage choice (6, 10, 35, 40). It is likely that stronger association of Lck with CD4 ensures greater availability of relative amount of fully activated Zap70 in MHCII-signaled thymocytes leading to stronger signal and consequent CD4 lineage choice. This also ensures rapid degradation of partially activated Zap70 that could interfere in signaling and promote CD8 lineage choice in MHCII-signaled cells. In contrast, weaker Lck association with CD8 (15, 44) would result in relatively fewer phospho-Zap70 available for signaling leading to signal disruption and CD8 lineage choice in MHCI-signaled thymocytes. A recent report describing FlipFlop mouse strongly support the signal duration model of CD4 and CD8 lineage choice (58). We predict that in FlipFlop mouse even though CD8 co-receptor encoded by the *Cd4* locus is weakly associated with Lck, its higher and continuous expression may result in accumulation of a sufficient amount of activated Lck, higher Zap70 activation and its rapid degradation leading to helper lineage choice, while the converse may happen with lower and disrupted CD4 co-receptor expression from the *Cd8* locus. Thus, cytotoxic lineage choice may be more susceptible to Zap70 amount, while helper lineage choice may be more affected by level of Lck activity (6). It may also explain why CD8 T cell, compared CD4 T cell, production is more severely affected by deficiency or hypomorphic expression of Zap70 in human patients (59–65).

Why would Lck, which promotes Zap70 activation critical for TCR signaling, negatively regulate Zap70 amount? Binding of Zap70 to phospho-CD3 promotes Zap70 activation due to partially destabilized auto-inhibition due to disruption of I-A linker and kinase domain interaction. This promotes Lck-mediated phosphorylation of Y315/Y319 resulting in complete relief from auto-inhibition leading to full Zap70 activation (46), which is required for its release from TCR complex for signal propagation upon engagement by p/MHC (56, 66). If Zap70 bound to phospho-CD3 is not phosphorylated it may act as a dominant negative (55), while undesired phosphorylation leading to its activation may result in autoimmunity due to increased sensitivity of TCR to self-ligands (28, 67, 68). Our data suggest any alteration in Zap70 conformation resulting in weakened auto-inhibition renders it unstable and this may be necessary to prevent undesired signal transduction. Indeed, W360P in the kinase domain and W163C mutation in the C-SH2 domain of Zap70 weakens its auto-inhibition resulting in open conformation and significantly lower protein expression and autoimmune disorders by different mechanism; W360P mutation results in hyperactive Zap70, whereas W163C mutated Zap70 is hypoactive due to its inability to bind to phospho-CD3 chains and subsequent lack of Lck-induced phosphorylation (65, 67, 69). That open conformation is detrimental to protein stability is also supported by reduced Zap70 expression in mice expressing YYAA mutated Zap70 leading to impaired TCR signaling and susceptibility to autoimmunity (70).

In conclusion, data reported here provide strong evidence that an open conformation of Zap70 induced by Lck-mediated phosphorylation of Y315/Y319 negatively impacts Zap70 stability in developing thymocytes and mature T cells, and forms the basis of differential Zap70 expression and activation in thymic subsets and mature CD4 and CD8 T cells.

## Data Availability Statement

The original contributions presented in the study are included in the article/**Supplementary Material**. Further inquiries can be directed to the corresponding author.

## Ethics Statement

Animal experimentation protocols were approved by the CRHMR animal care committee and performed in accordance with the Canadian Committee for Animal Care.

## Author Contributions

HD, CT, and MV performed experiments. VD and HD analyzed data. D-CR provided reagents and resources. VD conceptualized the study, and VD and D-CR supervised the study. All authors discussed and commented on manuscript. All authors contributed to the article and approved the submitted version.

## Conflict of Interest

The authors declare that the research was conducted in the absence of any commercial or financial relationships that could be construed as a potential conflict of interest.

## Publisher’s Note

All claims expressed in this article are solely those of the authors and do not necessarily represent those of their affiliated organizations, or those of the publisher, the editors and the reviewers. Any product that may be evaluated in this article, or claim that may be made by its manufacturer, is not guaranteed or endorsed by the publisher.

## Supplementary Material

Supplementary Figure 1 shows gating strategy for thymocytes and splenic T cells for evaluating Zap70 expression described in all the figures in the main text.

Supplementary Figure 2 Constitutively active Lck results in Zap70 diminution in P14-TCR transgenic mice. **(A)** shows CD24/TCR staining of total thymocytes and CD4/CD8 profile of mature thymocytes (CD24^lo^TCR^+^). Bar graph shows frequency of CD4^+^ and CD8^+^ thymocytes and CD4/CD8 ratio in P14 and P14^+^Lck^Y505F^ mice. **(B)** shows histogram of Zap70 staining in DP, CD4^+^CD8^lo^, CD4^+^ and CD8^+^ thymocytes from P14 mice expressing or not Lck^Y505F^ transgene. Number in the histogram represent MFI for Zap70 staining. Bar graph shows compilation of Zap70 MFI for the indicated thymic subset normalized to CD8 SP thymocytes from P14 control mice in each experiment. **(C)** TCR and CD4/CD8 staining and frequency of splenic T cells. Zap70 staining of CD4 and CD8 T cells from P14 mice expressing or not Lck^Y505F^ is shown. Bar graph shows compilation of Zap70 MFI in the indicated T cell subsets from P14 and P14^+^Lck^Y505F^ mice normalized to that in CD8 T cells from P14 mice in each experiment. Data are representative example of 3 or more independent experiments (n > 6 mice).

Supplementary Figure 3 Constitutive Thpok-H expression does not alter Zap70 expression. **(A)** Staining of thymocyte for the indicated surface markers from OTI and OTI^+^Th-H^+^ mice is shown. Zap70 staining histograms for DP, CD4^+^8^lo^, CD4^+^ and CD8^+^ thymocytes are shown. **(B)** shows TCR and CD4/CD8 staining of splenic T cells and shows analysis of Zap70 expression in CD4 and CD8 T cells from OTI and OTI^+^Th-H^+^ mice. Number in the histogram indicated Zap70 MFI (n>3 mice in three independent experiments).

## References

[1] Hogquist KA Jameson SC. The Self-Obsession of T Cells: How TCR Signaling Thresholds Affect Fate 'Decisions' and Effector Function. Nat Immunol (2014) 15(9):815–23. doi: 10.1038/ni.2938 PMC4348363 25137456

[2] Trobridge PA Forbush KA Levin SD. Positive and Negative Selection of Thymocytes Depends on Lck Interaction With the CD4 and CD8 Coreceptors. J Immunol (2001) 166(2):809–18. doi: 10.4049/jimmunol.166.2.809 11145654

[3] Taniuchi I. CD4 Helper and CD8 Cytotoxic T Cell Differentiation. Annu Rev Immunol (2018) 36:579–601. doi: 10.1146/annurev-immunol-042617-053411 29677476

[4] Gascoigne NR Rybakin V Acuto O Brzostek J. TCR Signal Strength and T Cell Development. Annu Rev Cell Dev Biol (2016) 32:327–48. doi: 10.1146/annurev-cellbio-111315-125324 27712102

[5] Klein L Kyewski B Allen PM Hogquist KA. Positive and Negative Selection of the T Cell Repertoire: What Thymocytes See (and Don't See). Nat Rev Immunol (2014) 14(6):377–91. doi: 10.1038/nri3667 PMC4757912 24830344

[6] Alarcon B van Santen HM. Two Receptors, Two Kinases, and T Cell Lineage Determination. Sci Signal (2010) 3(114):pe11. doi: 10.1126/scisignal.3114pe11 20332426

[7] Ashouri JF Lo WL Nguyen TTT Shen L Weiss A. ZAP70, Too Little, Too Much can Lead to Autoimmunity. Immunol Rev (2021) 37:145–60. doi: 10.1111/imr.13058 PMC8986586 34923645

[8] Fehling HJ Iritani BM Krotkova A Forbush KA Laplace C Perlmutter RM. Restoration of Thymopoiesis in pT Alpha-/- Mice by Anti-CD3epsilon Antibody Treatment or With Transgenes Encoding Activated Lck or Tailless pT Alpha. Immunity (1997) 6(6):703–14. doi: 10.1016/S1074-7613(00)80446-X 9208843

[9] Erman B Alag AS Dahle O van Laethem F Sarafova SD Guinter TI. Coreceptor Signal Strength Regulates Positive Selection But Does Not Determine CD4/CD8 Lineage Choice in a Physiologic *In Vivo* Model. J Immunol (2006) 177(10):6613–25. doi: 10.4049/jimmunol.177.10.6613 17082573

[10] Sohn SJ Forbush KA Pan XC Perlmutter RM. Activated P56lck Directs Maturation of Both CD4 and CD8 Single-Positive Thymocytes. J Immunol (2001) 166(4):2209–17. doi: 10.4049/jimmunol.166.4.2209 11160274

[11] Au-Yeung BB Shah NH Shen L Weiss A. ZAP-70 in Signaling, Biology, and Disease. Annu Rev Immunol (2018) 36:127–56. doi: 10.1146/annurev-immunol-042617-053335 PMC13317156 29237129

[12] Palacios EH Weiss A. Distinct Roles for Syk and ZAP-70 During Early Thymocyte Development. J Exp Med (2007) 204(7):1703–15. doi: 10.1084/jem.20070405 PMC2118636 17606633

[13] Itano A Salmon P Kioussis D Tolaini M Corbella P Robey E. The Cytoplasmic Domain of CD4 Promotes the Development of CD4 Lineage T Cells. J Exp Med (1996) 183(3):731–41. doi: 10.1084/jem.183.3.731 PMC2192343 8642277

[14] Irie HY Mong MS Itano A Crooks ME Littman DR Burakoff SJ. The Cytoplasmic Domain of CD8 Beta Regulates Lck Kinase Activation and CD8 T Cell Development. J Immunol (1998) 161(1):183–91. 9647223

[15] Wiest DL Yuan L Jefferson J Benveniste P Tsokos M Klausner RD. Regulation of T Cell Receptor Expression in Immature CD4+CD8+ Thymocytes by P56lck Tyrosine Kinase: Basis for Differential Signaling by CD4 and CD8 in Immature Thymocytes Expressing Both Coreceptor Molecules. J Exp Med (1993) 178(5):1701–12. doi: 10.1084/jem.178.5.1701 PMC2191226 8228817

[16] Yamaguchi H Hendrickson WA. Structural Basis for Activation of Human Lymphocyte Kinase Lck Upon Tyrosine Phosphorylation. Nature (1996) 384(6608):484–9. doi: 10.1038/384484a0 8945479

[17] Schoenborn JR Tan YX Zhang C Shokat KM Weiss A. Feedback Circuits Monitor and Adjust Basal Lck-Dependent Events in T Cell Receptor Signaling. Sci Signal (2011) 4(190):ra59. doi: 10.1126/scisignal.2001893 21917715 PMC4080844

[18] Ostergaard HL Shackelford DA Hurley TR Johnson P Hyman R Sefton BM. Expression of CD45 Alters Phosphorylation of the Lck-Encoded Tyrosine Protein Kinase in Murine Lymphoma T-Cell Lines. Proc Natl Acad Sci U S A (1989) 86(22):8959–63. doi: 10.1073/pnas.86.22.8959 PMC298410 2530588

[19] Nada S Yagi T Takeda H Tokunaga T Nakagawa H Ikawa Y. Constitutive Activation of Src Family Kinases in Mouse Embryos That Lack Csk. Cell (1993) 73(6):1125–35. doi: 10.1016/0092-8674(93)90642-4 8513497

[20] Mustelin T Coggeshall KM Altman A. Rapid Activation of the T-Cell Tyrosine Protein Kinase Pp56lck by the CD45 Phosphotyrosine Phosphatase. Proc Natl Acad Sci U S A (1989) 86(16):6302–6. doi: 10.1073/pnas.86.16.6302 PMC297826 2548204

[21] Schmedt C Saijo K Niidome T Kuhn R Aizawa S Tarakhovsky A. Csk Controls Antigen Receptor-Mediated Development and Selection of T-Lineage Cells. Nature (1998) 394(6696):901–4. doi: 10.1038/29802 9732874

[22] Bergman M Mustelin T Oetken C Partanen J Flint NA Amrein KE. The Human P50csk Tyrosine Kinase Phosphorylates P56lck at Tyr-505 and Down Regulates its Catalytic Activity. EMBO J (1992) 11(8):2919–24. doi: 10.1002/j.1460-2075.1992.tb05361.x PMC556773 1639064

[23] Courtney AH Shvets AA Lu W Griffante G Mollenauer M Horkova V. CD45 Functions as a Signaling Gatekeeper in T Cells. Sci Signal (2019) 12(604). doi: 10.1126/scisignal.aaw8151 PMC6948007 31641081

[24] van Oers NS Killeen N Weiss A. Lck Regulates the Tyrosine Phosphorylation of the T Cell Receptor Subunits and ZAP-70 in Murine Thymocytes. J Exp Med (1996) 183(3):1053–62. doi: 10.1084/jem.183.3.1053 PMC2192313 8642247

[25] Wange RL Malek SN Desiderio S Samelson LE. Tandem SH2 Domains of ZAP-70 Bind to T Cell Antigen Receptor Zeta and CD3 Epsilon From Activated Jurkat T Cells. J Biol Chem (1993) 268(26):19797–801. doi: 10.1016/S0021-9258(19)36584-6 8366117

[26] Gascoigne NR Casas J Brzostek J Rybakin V. Initiation of TCR Phosphorylation and Signal Transduction. Front Immunol (2011) 2:72. doi: 10.3389/fimmu.2011.00072 22566861 PMC3342367

[27] Iwashima M Irving BA van Oers NS Chan AC Weiss A. Sequential Interactions of the TCR With Two Distinct Cytoplasmic Tyrosine Kinases. Science (1994) 263(5150):1136–9. doi: 10.1126/science.7509083 7509083

[28] Chan AC Kadlecek TA Elder ME Filipovich AH Kuo WL Iwashima M. ZAP-70 Deficiency in an Autosomal Recessive Form of Severe Combined Immunodeficiency. Science (1994) 264(5165):1599–601. doi: 10.1126/science.8202713 8202713

[29] Negishi I Motoyama N Nakayama K Nakayama K Senju S Hatakeyama S. Essential Role for ZAP-70 in Both Positive and Negative Selection of Thymocytes. Nature (1995) 376(6539):435–8. doi: 10.1038/376435a0 7630421

[30] Singer A Adoro S Park JH. Lineage Fate and Intense Debate: Myths, Models and Mechanisms of CD4- Versus CD8-Lineage Choice. Nat Rev Immunol (2008) 8(10):788–801. doi: 10.1038/nri2416 18802443 PMC2760737

[31] Hernandez-Hoyos G Sohn SJ Rothenberg EV Alberola-Ila J. Lck Activity Controls CD4/CD8 T Cell Lineage Commitment. Immunity (2000) 12(3):313–22. doi: 10.1016/S1074-7613(00)80184-3 10755618

[32] Bosselut R. CD4/CD8-Lineage Differentiation in the Thymus: From Nuclear Effectors to Membrane Signals. Nat Rev Immunol (2004) 4(7):529–40. doi: 10.1038/nri1392 15229472

[33] Hwang S Palin AC Li L Song KD Lee J Herz J. TCR ITAM Multiplicity is Required for the Generation of Follicular Helper T-Cells. Nat Commun (2015) 6:6982. doi: 10.1038/ncomms7982 25959494 PMC4428620

[34] Love PE Shores EW Johnson MD Tremblay ML Lee EJ Grinberg A. T Cell Development in Mice That Lack the Zeta Chain of the T Cell Antigen Receptor Complex. Science (1993) 261(5123):918–21. doi: 10.1126/science.7688481 7688481

[35] Saini M Sinclair C Marshall D Tolaini M Sakaguchi S Seddon B. Regulation of Zap70 Expression During Thymocyte Development Enables Temporal Separation of CD4 and CD8 Repertoire Selection at Different Signaling Thresholds. Sci Signal (2010) 3(114):ra23. doi: 10.1126/scisignal.2000702 20332428

[36] Cheng AM Negishi I Anderson SJ Chan AC Bolen J Loh DY. The Syk and ZAP-70 SH2-Containing Tyrosine Kinases are Implicated in Pre-T Cell Receptor Signaling. Proc Natl Acad Sci U S A (1997) 94(18):9797–801. doi: 10.1073/pnas.94.18.9797 PMC23271 9275205

[37] Cosgrove D Gray D Dierich A Kaufman J Lemeur M Benoist C. Mice Lacking MHC Class II Molecules. Cell (1991) 66(5):1051–66. doi: 10.1016/0092-8674(91)90448-8 1909605

[38] Riberdy JM Mostaghel E Doyle C. Disruption of the CD4-Major Histocompatibility Complex Class II Interaction Blocks the Development of CD4(+) T Cells *In Vivo* . Proc Natl Acad Sci U.S.A. (1998) 95(8):4493–8. doi: 10.1073/pnas.95.8.4493 PMC22517 9539765

[39] Wiest DL Ashe JM Abe R Bolen JB Singer A. TCR Activation of ZAP70 is Impaired in CD4+CD8+ Thymocytes as a Consequence of Intrathymic Interactions That Diminish Available P56lck. Immunity (1996) 4(5):495–504. doi: 10.1016/S1074-7613(00)80415-X 8630734

[40] Zeidan N Damen H Roy DC Dave VP. Critical Role for TCR Signal Strength and MHC Specificity in ThPOK-Induced CD4 Helper Lineage Choice. J Immunol (2019) 202(11):3211–25. doi: 10.4049/jimmunol.1801464 31036767

[41] Wei Q Brzostek J Sankaran S Casas J Hew LS Yap J. Lck Bound to Coreceptor is Less Active Than Free Lck. Proc Natl Acad Sci U S A (2020) 117(27):15809–17. doi: 10.1073/pnas.1913334117 PMC7355011 32571924

[42] Gudipati V Rydzek J Doel-Perez I Goncalves VDR Scharf L Konigsberger S. Inefficient CAR-Proximal Signaling Blunts Antigen Sensitivity. Nat Immunol (2020) 21(8):848–56. doi: 10.1038/s41590-020-0719-0 32632291

[43] Penna D Muller S Martinon F Demotz S Iwashima M Valitutti S. Degradation of ZAP-70 Following Antigenic Stimulation in Human T Lymphocytes: Role of Calpain Proteolytic Pathway. J Immunol (1999) 163(1):50–6. 10384098

[44] Veillette A Zuniga-Pflucker JC Bolen JB Kruisbeek AM. Engagement of CD4 and CD8 Expressed on Immature Thymocytes Induces Activation of Intracellular Tyrosine Phosphorylation Pathways. J Exp Med (1989) 170(5):1671–80. doi: 10.1084/jem.170.5.1671 PMC2189493 2478653

[45] Thill PA Weiss A Chakraborty AK. Phosphorylation of a Tyrosine Residue on Zap70 by Lck and Its Subsequent Binding *via* an SH2 Domain May Be a Key Gatekeeper of T Cell Receptor Signaling *In Vivo* . Mol Cell Biol (2016) 36(18):2396–402. doi: 10.1128/MCB.00165-16 PMC5007795 27354065

[46] Yan Q Barros T Visperas PR Deindl S Kadlecek TA Weiss A. Structural Basis for Activation of ZAP-70 by Phosphorylation of the SH2-Kinase Linker. Mol Cell Biol (2013) 33(11):2188–201. doi: 10.1128/MCB.01637-12 PMC3648074 23530057

[47] Hsu LY Tan YX Xiao Z Malissen M Weiss A. A Hypomorphic Allele of ZAP-70 Reveals a Distinct Thymic Threshold for Autoimmune Disease Versus Autoimmune Reactivity. J Exp Med (2009) 206(11):2527–41. doi: 10.1084/jem.20082902 PMC2768860 19841086

[48] Brdicka T Kadlecek TA Roose JP Pastuszak AW Weiss A. Intramolecular Regulatory Switch in ZAP-70: Analogy With Receptor Tyrosine Kinases. Mol Cell Biol (2005) 25(12):4924–33. doi: 10.1128/MCB.25.12.4924-4933.2005 PMC1140569 15923611

[49] Magnan A Di Bartolo V Mura AM Boyer C Richelme M Lin YL. T Cell Development and T Cell Responses in Mice With Mutations Affecting Tyrosines 292 or 315 of the ZAP-70 Protein Tyrosine Kinase. J Exp Med (2001) 194(4):491–505. doi: 10.1084/jem.194.4.491 11514605 PMC2193493

[50] Gong Q Jin X Akk AM Foger N White M Gong G. Requirement for Tyrosine Residues 315 and 319 Within Zeta Chain-Associated Protein 70 for T Cell Development. J Exp Med (2001) 194(4):507–18. doi: 10.1084/jem.194.4.507 PMC2193491 11514606

[51] Veillette A Bookman MA Horak EM Bolen JB. The CD4 and CD8 T Cell Surface Antigens are Associated With the Internal Membrane Tyrosine-Protein Kinase P56lck. Cell (1988) 55(2):301–8. doi: 10.1016/0092-8674(88)90053-0 3262426

[52] Lo WL Shah NH Ahsan N Horkova V Stepanek O Salomon AR. Lck Promotes Zap70-Dependent LAT Phosphorylation by Bridging Zap70 to LAT. Nat Immunol (2018) 19(7):733–41. doi: 10.1038/s41590-018-0131-1 PMC6202249 29915297

[53] D'Oro U Vacchio MS Weissman AM Ashwell JD. Activation of the Lck Tyrosine Kinase Targets Cell Surface T Cell Antigen Receptors for Lysosomal Degradation. Immunity (1997) 7(5):619–28. doi: 10.1016/S1074-7613(00)80383-0 9390686

[54] van Oers NS Killeen N Weiss A. ZAP-70 is Constitutively Associated With Tyrosine-Phosphorylated TCR Zeta in Murine Thymocytes and Lymph Node T Cells. Immunity (1994) 1(8):675–85. doi: 10.1016/1074-7613(94)90038-8 7600293

[55] Qian D Mollenauer MN Weiss A. Dominant-Negative Zeta-Associated Protein 70 Inhibits T Cell Antigen Receptor Signaling. J Exp Med (1996) 183(2):611–20. doi: 10.1084/jem.183.2.611 PMC2192449 8627172

[56] Katz ZB Novotna L Blount A Lillemeier BF. A Cycle of Zap70 Kinase Activation and Release From the TCR Amplifies and Disperses Antigenic Stimuli. Nat Immunol (2017) 18(1):86–95. doi: 10.1038/ni.3631 27869819 PMC5490839

[57] Davey GM Schober SL Endrizzi BT Dutcher AK Jameson SC Hogquist KA. Preselection Thymocytes are More Sensitive to T Cell Receptor Stimulation Than Mature T Cells. J Exp Med (1998) 188(10):1867–74. doi: 10.1084/jem.188.10.1867 PMC2212399 9815264

[58] Shinzawa M Moseman EA Gossa S Mano Y Bhattacharya A Guinter T. Reversal of the T Cell Immune System Reveals the Molecular Basis for T Cell Lineage Fate Determination in the Thymus. Nat Immunol (2022) 23(5):731–42. doi: 10.1038/s41590-022-01187-1 PMC9098387 35523960

[59] Elder ME Skoda-Smith S Kadlecek TA Wang F Wu J Weiss A. Distinct T Cell Developmental Consequences in Humans and Mice Expressing Identical Mutations in the DLAARN Motif of ZAP-70. J Immunol (2001) 166(1):656–61. doi: 10.4049/jimmunol.166.1.656 11123350

[60] Elder ME Lin D Clever J Chan AC Hope TJ Weiss A. Human Severe Combined Immunodeficiency Due to a Defect in ZAP-70, a T Cell Tyrosine Kinase. Science (1994) 264(5165):1596–9. doi: 10.1126/science.8202712 8202712

[61] Elder ME Hope TJ Parslow TG Umetsu DT Wara DW Cowan MJ. Severe Combined Immunodeficiency With Absence of Peripheral Blood CD8+ T Cells Due to ZAP-70 Deficiency. Cell Immunol (1995) 165(1):110–7. doi: 10.1006/cimm.1995.1193 7671314

[62] Arpaia E Shahar M Dadi H Cohen A Roifman CM. Defective T Cell Receptor Signaling and CD8+ Thymic Selection in Humans Lacking Zap-70 Kinase. Cell (1994) 76(5):947–58. doi: 10.1016/0092-8674(94)90368-9 8124727

[63] Picard C Dogniaux S Chemin K Maciorowski Z Lim A Mazerolles F. Hypomorphic Mutation of ZAP70 in Human Results in a Late Onset Immunodeficiency and No Autoimmunity. Eur J Immunol (2009) 39(7):1966–76. doi: 10.1002/eji.200939385 19548248

[64] Sharifinejad N Jamee M Zaki-Dizaji M Lo B Shaghaghi M Mohammadi H. Clinical, Immunological, and Genetic Features in 49 Patients With ZAP-70 Deficiency: A Systematic Review. Front Immunol (2020) 11:831. doi: 10.3389/fimmu.2020.00831 32431715 PMC7214800

[65] Chan AY Punwani D Kadlecek TA Cowan MJ Olson JL Mathes EF. A Novel Human Autoimmune Syndrome Caused by Combined Hypomorphic and Activating Mutations in ZAP-70. J Exp Med (2016) 213(2):155–65. doi: 10.1084/jem.20150888 PMC4749924 26783323

[66] Williams BL Irvin BJ Sutor SL Chini CC Yacyshyn E Bubeck Wardenburg J. Phosphorylation of Tyr319 in ZAP-70 is Required for T-Cell Antigen Receptor-Dependent Phospholipase C-Gamma1 and Ras Activation. EMBO J (1999) 18(7):1832–44. doi: 10.1093/emboj/18.7.1832 PMC1171269 10202147

[67] Shen L Matloubian M Kadlecek TA Weiss A. A Disease-Associated Mutation That Weakens ZAP70 Autoinhibition Enhances Responses to Weak and Self-Ligands. Sci Signal (2021) 14(668). doi: 10.1126/scisignal.abc4479 PMC8009134 33531381

[68] Ashouri JF Lo WL Nguyen TTT Shen L Weiss A. TZAP70, Too Little, Too Much can Lead to Autoimmunity. Immunol Rev (2022) 307(1):145–60. doi: 10.1111/imr.13058 PMC8986586 34923645

[69] Sakaguchi N Takahashi T Hata H Nomura T Tagami T Yamazaki S. Altered Thymic T-Cell Selection Due to a Mutation of the ZAP-70 Gene Causes Autoimmune Arthritis in Mice. Nature (2003) 426(6965):454–60. doi: 10.1038/nature02119 14647385

[70] Hsu LY Cheng DA Chen Y Liang HE Weiss A. Destabilizing the Autoinhibitory Conformation of Zap70 Induces Up-Regulation of Inhibitory Receptors and T Cell Unresponsiveness. J Exp Med (2017) 214(3):833–49. doi: 10.1084/jem.20161575 PMC5339679 28159798

