# Supplementary figures and images for: Negative Regulation of Zap70 by Lck Forms the Mechanistic Basis of Differential Expression in CD4 and CD8 T Cells
Source: Front Immunol. 2022 Jul 4;13:935367. doi: 10.3389/fimmu.2022.935367 (PMC9289233; doi:10.3389/fimmu.2022.935367)

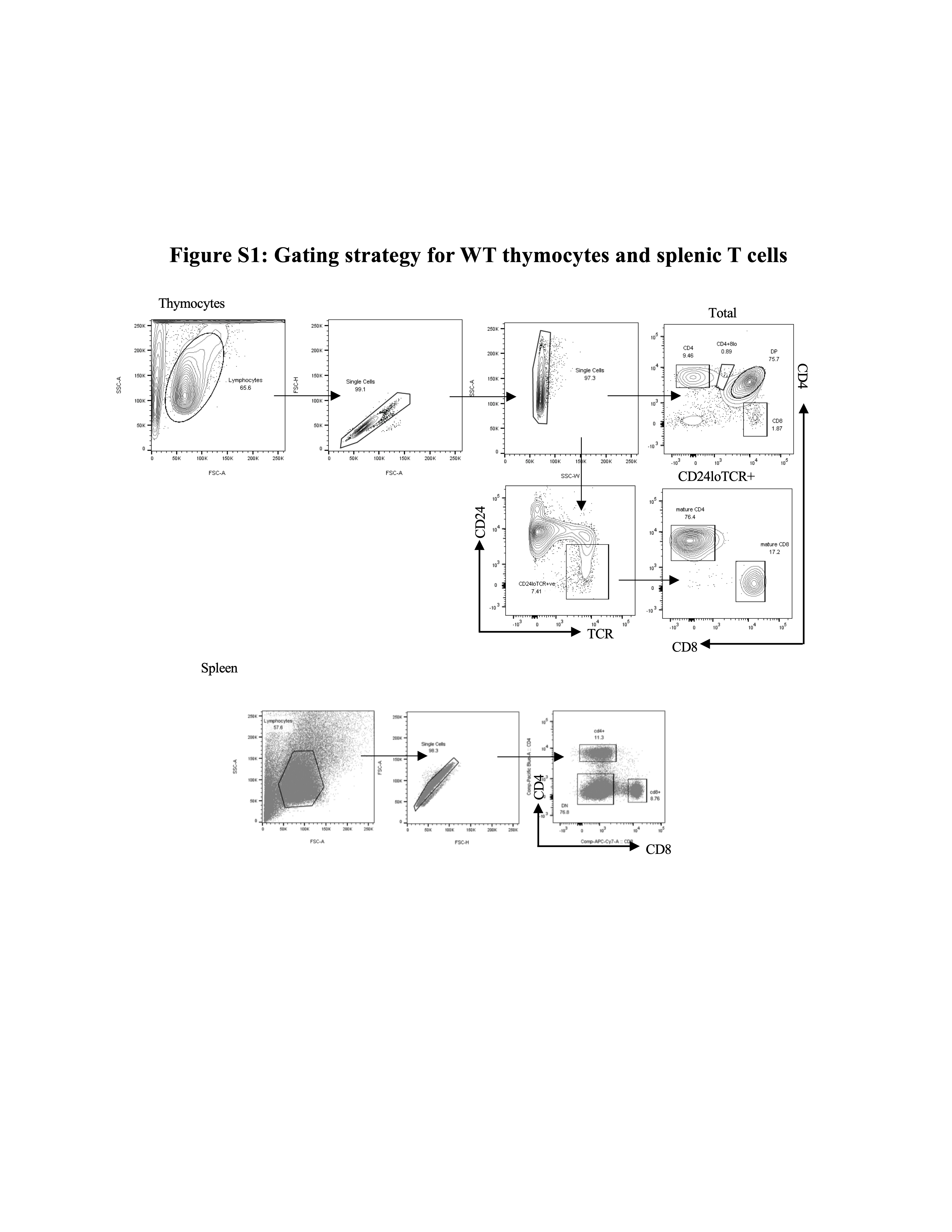

Supplement: Supplementary Figure 1 — shows gating strategy for thymocytes and splenic T cells for evaluating Zap70 expression described in all the figures in the main text. [file Image_1.jpg]

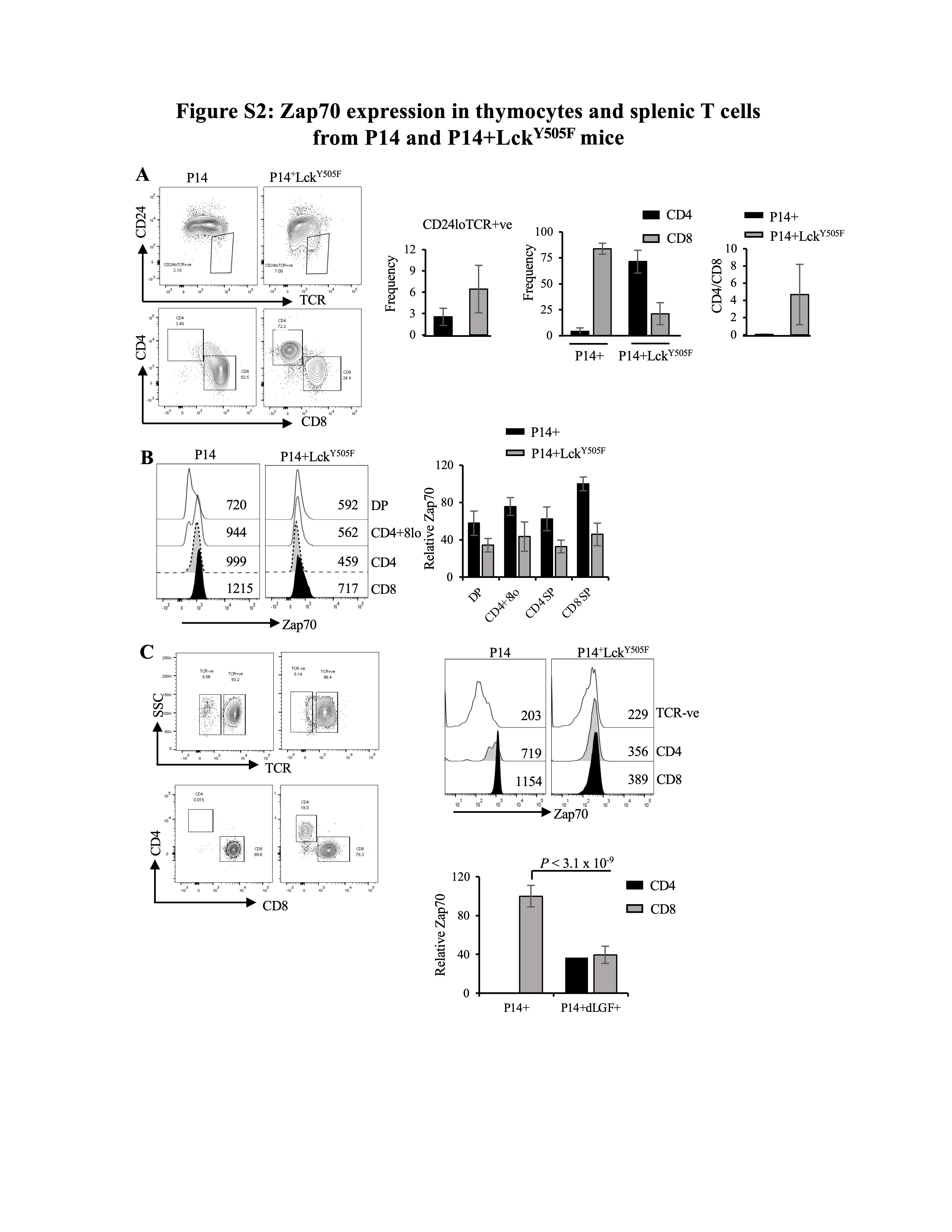

Supplement: Supplementary Figure 2 — Constitutively active Lck results in Zap70 diminution in P14-TCR transgenic mice. (A) shows CD24/TCR staining of total thymocytes and CD4/CD8 profile of mature thymocytes (CD24loTCR+). Bar graph shows frequency of CD4+ and CD8+ thymocytes and CD4/CD8 ratio in P14 and P14+LckY505F mice. (B) shows histogram of Zap70 staining in DP, CD4+CD8lo, CD4+ and CD8+ thymocytes from P14 mice expressing or not LckY505F transgene. Number in the histogram represent MFI for Zap70 staining. Bar graph shows compilation of Zap70 MFI for the indicated thymic subset normalized to CD8 SP thymocytes from P14 control mice in each experiment. (C) TCR and CD4/CD8 staining and frequency of splenic T cells. Zap70 staining of CD4 and CD8 T cells from P14 mice expressing or not LckY505F is shown. Bar graph shows compilation of Zap70 MFI in the indicated T cell subsets from P14 and P14+LckY505F mice normalized to that in CD8 T cells from P14 mice in each experiment. Data are representative example of 3 or more independent experiments (n > 6 mice). [file Image_2.jpg]

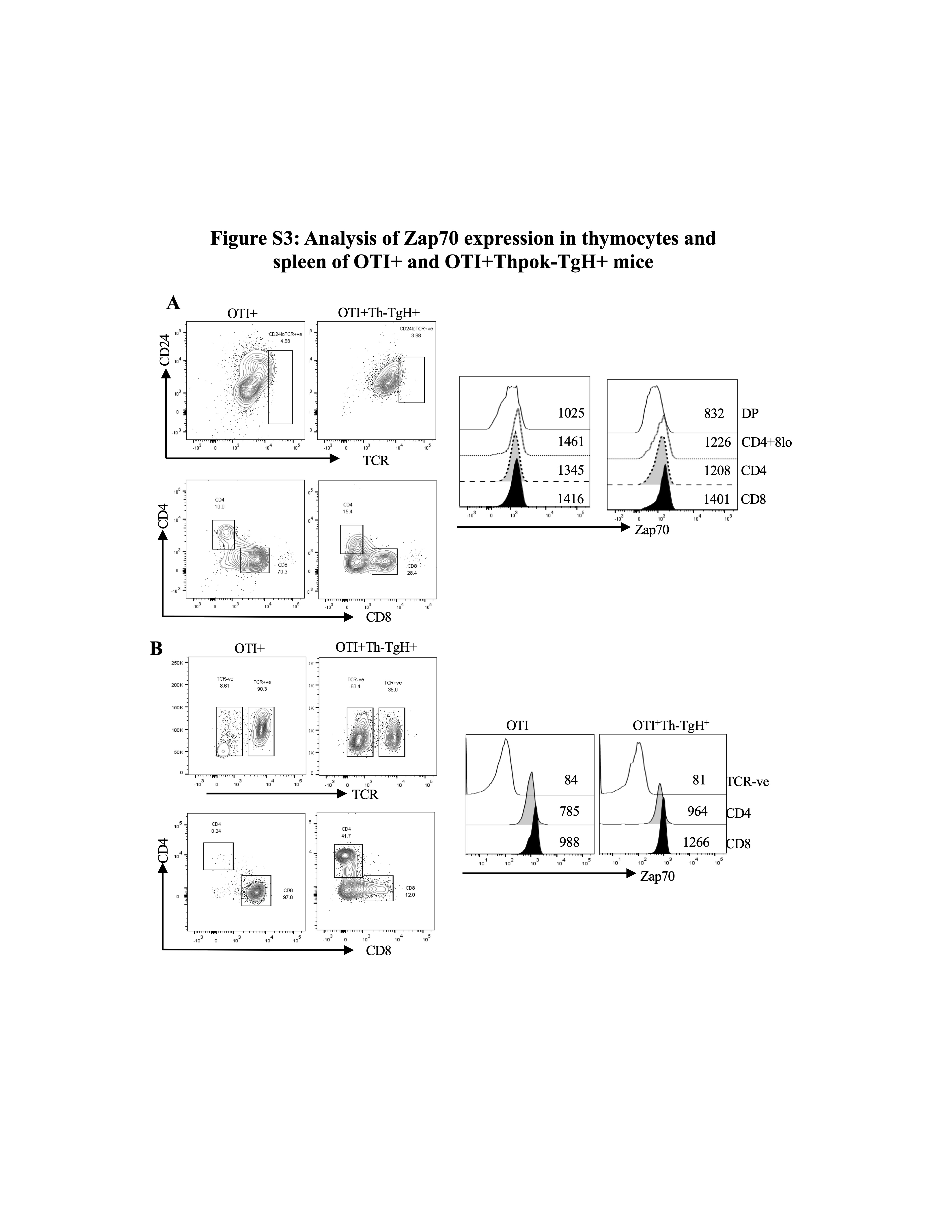

Supplement: Supplementary Figure 3 — Constitutive Thpok-H expression does not alter Zap70 expression. (A) Staining of thymocyte for the indicated surface markers from OTI and OTI+Th-H+ mice is shown. Zap70 staining histograms for DP, CD4+8lo, CD4+ and CD8+ thymocytes are shown. (B) shows TCR and CD4/CD8 staining of splenic T cells and shows analysis of Zap70 expression in CD4 and CD8 T cells from OTI and OTI+Th-H+ mice. Number in the histogram indicated Zap70 MFI (n>3 mice in three independent experiments). [file Image_3.jpg]
